# Association between lifestyle factors and suboptimal health status among Chinese college freshmen: a cross-sectional study

**DOI:** 10.1186/s12889-017-5002-4

**Published:** 2018-01-05

**Authors:** Chenjin Ma, Wangli Xu, Long Zhou, Shuangge Ma, Yu Wang

**Affiliations:** 1 Center for Applied Statistics and School of Statistics, Renmin University of China, Beijing, China; 2 School of Public Health, Yale University, New Haven, CT USA

**Keywords:** Suboptimal health status, Lifestyle behaviors, Electronic devices, College freshmen, China

## Abstract

**Background:**

Suboptimal health status (SHS) is the third state between good health and disease. SHS is the clinical or pre-disease status of psychosomatic disease and a major global public health challenge. Although its underlying causes remain unclear, lifestyle is one of the most important factors affecting health status.

**Methods:**

A cross-sectional survey was conducted at Renmin University of China in September of 2015. Data were collected from college freshmen using a questionnaire covering characteristics, lifestyle, nutrition status, and health status. A total of 6025 questionnaires were distributed during the study period, and 5344 completed responses were received.

**Results:**

The prevalence rates for the “healthy,” “SHS,” and “disease” groups of college freshmen were 46.7% (2433), 51.2% (2667), and 2.1% (111), respectively. It is notable that health status was significantly positively correlated with lifestyle (Spearman’s *r* = 0.4435, *p* < 0.001). The multivariate Logistic regression results showed that students who were relatively younger and students from rural areas had a higher percentage of SHS. Good sleep quality (aOR = 0.650, 95%CI = 0.612–0.690), abundant physical exercise (aOR = 0.889, 95%CI = 0.845–0.933), and adequate nutrition intake (aOR = 0.868, 95%CI = 0.864–0.908) are negatively associated with SHS. Overuse of electronic devices (aOR = 1.066, 95%CI = 1.013–1.121), smoking (aOR = 1.824, 95%CI = 1.195–2.755), and weight loss (aOR = 1.255, 95%CI = 1.043–1.509) are positively associated with SHS.

**Conclusions:**

Poor lifestyle behaviors are associated with SHS. In particular, the overuse of electronic devices is one of underlying causes of SHS. By altering lifestyle behaviors for the better, the health statuses of these college freshmen can be effectively improved.

**Electronic supplementary material:**

The online version of this article (10.1186/s12889-017-5002-4) contains supplementary material, which is available to authorized users.

## Background

In 1946, the World Health Organization (WHO) defined health as “a state of complete physical, mental, and social well-being and not merely the absence of disease or infirmity” [1, 2]. With changes in living environments, especially urbanization, more and more people reported suboptimal health without a diagnosable condition, which is called suboptimal health status (SHS) [3]. SHS is an intermediate state between health and disease, which is often medically undiagnosed. In the traditional Chinese medicine guidelines released by the China Association of Chinese Medicine (CACM), medically undiagnosed or functional somatic syndromes [4–6] are characterized by a decline in vitality, in physiological function, and in the capacity for adaptation [3, 7]. SHS people frequently suffer symptoms, such as headaches, dizziness, chronic fatigue, depression, anxiety, functional system disorders (e.g., disorders of the digestive system, cardiovascular system, urinary system, etc.) and non-specific pain (e.g., back pain and chest pain). Accordingly, SHS sufferers often experienced impaired quality of life, frequent hospital visits, and expensive medical expenses [6, 7].

Recent years, many other countries widely accepted the concept of SHS, including Japan, Canada, and Australia [8–10]. Surveys of SHS have involved people of different groups such as teachers, civil servants, businessmen, community residents, medical personnel, and others [3, 11–13]. Due to the inconsistent definitions of SHS adopted by these different studies, as well as the different questionnaires or scales used, the reported rates of SHS vary greatly, from 20 to 80% [3, 10, 14]. In 1998, some researchers conducted a thorough examination of 6000 asymptomatic “healthy people.” The results showed that 72.8% were in the “suboptimal health status” range [15]. The incidence of SHS is high, but its causes are unclear. According to the previous studies, lifestyle behaviors are considered as one of the most important factors affecting health [16–19], and poor lifestyle factors may be associated with SHS, such as work-related and study-related stress, physical inactivity, short sleep time and unhealthy diet patterns [3, 10, 14, 20, 21].

Most previous surveys on suboptimal health status have mainly focused on specific populations, such as teachers, civil servants, etc. [11, 15, 22]. Only a few studies have explored SHS among university students, who are generally considered to be a relatively healthy population. Some studies, though, have showed that the rate of SHS in this population is high. One study of 11,144 students in 2013 revealed that the prevalence rate for the SHS group of respondents was 55.9%, and girls experienced a higher rate of SHS than boys [10]. For college students, particularly freshmen, their lifestyle behaviors can undergo great changes during campus life. Most typically, due to heavy study loads and anxiety, many students do not eat regularly, get sufficient sleep, or exercise adequately. As a result, they may suffer from headaches, insomnia, fatigue, and/or forgetfulness. Additionally, according to some global health behavior studies among university students, students had a high proportion of poor health behavior practices, and several health risk behaviors were identified, including overweight, poor dental practices, poor dietary patterns, tobacco use and sleeping habits. [23, 24] One cross-sectional survey of 800 university students in India found that there was a high rate of overweight and obesity and poor dietary patterns. [25] With the development of technology, more and more college students are using electronic devices. Researchers have begun to pay increased attention to the impact of electronic equipment on the health of students. A retrospective, nested, case-control study conducted from 2009 to 2011 showed that freshmen exhibiting signs of depression, learning maladaptation, and dissatisfaction could be an important target intervention population for reducing Internet addiction [26]. But no studies to date have focused on the relationship between the usage of electronic products and suboptimal health status. In order to explore the association between various lifestyle factors and suboptimal health status and fill the noted gaps in the research, we conducted a cross-sectional study among college freshmen in China.

## Methods

### Sample and data collection

A cross-sectional survey was conducted at Renmin University of China during September of 2015. The study sample included all freshmen enrolled in the university in 2015. The data were collected using a self-administered questionnaire. At the beginning of each survey, the interviewer introduced the nature of the survey. Each interviewee was asked to sign an informed consent form. An interviewee was excluded if he/she refused to participate. The questionnaire was completed by each student within 30 min. All data were kept strictly confidential. The study was approved by the ethics committee of Renmin University.

A total of 6025 questionnaires were distributed during the study period, and 5646 (93.7%) questionnaires were returned. A total of 5233 completed responses were analyzed in this study after a review, yielding a valid response rate of 86.5%.

The questionnaire (see Additional file 1) was composed of four sections: students’ characteristics, a health promotion lifestyle scale (HPLS), the Suboptimal Health Measurement ScaleV1.0 (SHMS V1.0), and history of diseases. The sections on students’ characteristics, the HPLS, and history of diseases were self-designed according to the questionnaires in other research works [27–32]. The SHMS V1.0 is a standardized questionnaire [33] used to assess respondents’ health status, and it contains a multidimensional, self-report symptom inventory developed by a research group in China [10, 21]. The SHMS V1.0 consists of 39 items in total, 35 of which are divided among three symptom dimensions (physiological symptoms: 14 items, psychological symptoms: 12 items, and social symptoms: 9 items), as indicated in Table 1. The remaining four items focus on health self-evaluation. For each item, there are five response categories (1 = never, 2 = occasionally, 3 = sometimes, 4 = constantly, and 5 = always).

**Table 1:** Structure Framework of the Suboptimal Health Measurement Scale V1.0

Dimension	Factors	Items
Physiological	Physical condition	3
	Organ function	6
	Body movement function	3
	Vigor	2
Psychological	Positive emotion	4
	Psychological symptoms	6
	Cognitive function	2
Social	Social adjustment	4
	Social resources	3
	Social support	2
Health self-evaluation	Physiological/Psychological/Social/Total	4
Total		39

### SHS evaluation

The evaluation of SHS in this study was performed according to the clinical guidelines for SHS published by the CACM [33]. Participants were asked about uncomfortable symptoms they experienced during the previous six months. The total scores were then calculated. The original score of every factor was equivalent to the total score of items included in this factor, and the original score of every dimension was equivalent to the total score of factors included. The original total score was equal to the sum of the three dimension scores. Then the original raw score was converted to obtain the final score according to the following formula for the conversion of original raw scores in dimension, subscale, and scale into percentile scores. The converted scores were used to analyze the outcomes—that is, the total score of health status, which ranges from 0~100. A lower total score represents a lower estimate of SHS.

Before completing the survey, the participating students each underwent a school health examination in a hospital. The health examination included a detailed medical history, a physical examination, blood hematology and biochemistry analyses, rest electrocardiogram, and chest radiography. Students with abnormal results were required to receive a reexamination. A total of 111 students (2.1%) were found to be in disease status. We deleted these data of these students in disease status, so the health status in this research was divided into two statuses: healthy and suboptimal health. Taking the unilateral P10 point of all dimensions of a crowd as the criterion, the dividing line scores of the three dimensions of physical suboptimal health, psychological suboptimal health, and social suboptimal health were 66.07, 52.08, and 55.56, respectively. When the score for any dimension of the three was lower than the dividing line score, it could be judged as suboptimal health status. If participants did not have SHS with respect to any of these three dimensions, they were considered healthy. The reliability of SHMS V1.0 has been confirmed, with a Cronbach’s α of 0.679.

### Lifestyle behaviors assessment

The health promotion lifestyle scale (HPLS) in this questionnaire was created on the basis of the “Health Promoting Lifestyle Profile (HPLP)” developed by Walker et al. [27–29] and the Pittsburgh Sleep Quality Index developed by Buysseet et al. [30–32]. Included were seven dimensions, constituting a total of 26 items: “sleep quality index” (7 items), “physical activity” (4 items), “usage of electronic devices” (3 items), “usage of tobacco” (1 item), “usage of alcohol” (1 item), “nutrition status” (9 items), and “losing weight or not” (1 item). All of these items over a period of the previous six months were investigated.

Sleep quality index (SQI) included sleep quality, sleep latency, sleep duration, and habitual sleep efficiency. Each item in the sleep quality index had scores of zero to three points, and the sum of the items was the total score for the SQI. The total scores ranged from zero to 12. The higher the score, the better a participant’s sleep quality. The scores for nutrition status range from 0~27, with a lower score representing a poorer nutrition status. The scores for the usage of electronic devices range from zero to 11, with a lower score signaling a less desirable behavior. The scores for “usage of alcohol,” “usage of tobacco,” and “losing weight or not” are zero or one, with zero representing “does not have this behavior” and one “has this behavior.”

### Statistical analysis

The statistical analysis was conducted using R software. The variables of normal distribution were described by mean and standard deviation, the variables of abnormal distribution were described by median and quartile, and categorical variables were described by frequency. Pearson *χ*^2^test and *t* test were used to compare variables, and the corresponding 95% confidence intervals (CIs) were calculated. *P*-value of <0.05 was considered to be significant for all tests. Logistic regression analysis was conducted, and the odds ratios (OR) and their significance levels were computed. All of these analyses were conducted on all of participants’ data except for those participants marked as “disease status”.

## Results

### Students’ characteristics

The prevalence rates for the healthy, SHS, and disease groups of college freshmen were 46.7% (2433), 51.2% (2667), and 2.1% (111), respectively. The characteristics of all participants (except for those in the disease status category) are presented in Table 2. Among the notable characteristics, 3426 (64.3%) were female, and the mean age was 21.7 years old, ranging from 16 to 45.

**Table 2:** Characteristics of Participating Students in the Survey (*n* = 5100)^a^

Participant Characteristics	n (%) or mean ± SD
Age in Years	21.7 ± 3.6
Age Group (years)	
15–19	2261 (44.3)
20–24	2164 (42.4)
25 and above	675 (13.3)
Gender	
Male	1768 (34.7)
Female	3321 (65.3)
Ethnicity	
Han	4606 (90.9)
Other	460 (9.1)
Student Type	
Bachelor’s candidates	2253 (44.3)
Master’s candidates	2361 (46.4)
Doctoral candidates	475 (9.3)
Area	
Rural	1309 (25.9)
Urban	3751 (74.1)
Health Status (range from 0–100)	73.13 ± 9.47
Health	2728 (52.4)
Suboptimal health	2481 (47.6)
Physiological Health Status (range from 0–100)	78.79 ± 9.69
Health	4586 (90.7)
Suboptimal health	468 (9.2)
Psychological Health Status (range from 0–100)	67.57 ± 12.10
Health	4640 (91.7)
Suboptimal health	4422 (8.3)
Social Health Status (range from 0–100)	71.25 ± 12.49
Health	4417 (87.2)
Suboptimal health	646 (12.8)
Sleep Quality Index (range from 0 to 12)	9.75 ± 1.49
Physical Activity (range from 0 to 12)	5.16 ± 1.92
Usage of Electronic Devices (range from 0 to 11)	6.59 ± 1.86
Nutrition Status (range from 0–27)	17.96 ± 3.16
Usage of Tobacco	
No	4895 (96.0)
Yes	201 (4.0)
Usage of Alcohol	
No	2820 (55.3)
Yes	2276 (44.7)
Losing Weight	
No	2425 (47.7)
Yes	2663 (52.3)

^a^Total may not add up to 5100 due to missing data

### Health promotion lifestyle scale

Table 3 presents the scores for the HPLS under two health statuses. The results showed that the scores for “sleep quality index,” “physical activity,” and “nutrition status” among healthy students (8.58 ± 1.77, 4.79 ± 2.10, 18.34 ± 3.02, respectively) were significantly higher than the scores among students classified with suboptimal health status (7.76 ± 1.86, 3.94 ± 2.16, 16.89 ± 3.28, respectively). This means that the sleep quality and nutrition status of the healthy students are better than that of the suboptimal health students, and healthy people more actively participate in physical exercise. Also, the score for “use of electronic devices” among healthy students were significantly lower than the scores among students classified with suboptimal health status (4.30 ± 1.74 vs 4.83 ± 2.03). It means suboptimal health people are more likely to be heavy users of electronic devices. It was further found that male students were more likely to be classified with SHS (*P* = 0.035).

**Table 3:** Scores of the Health Promotion Lifestyle Scale Under Two Health Statuses (n = 5100)^a^

Characteristics	Health Promotion Lifestyle Scale Scores		*P-*Value
	Healthy	SHS	
Age Group (years)			0.880
15–19	1084 (48.0%)	1175 (52.0%)	
20–24	1030 (47.6%)	1134 (52.4%)	
25 and above	317 (47.0%)	358 (53.0%)	
Gender			0.035
Male	821 (46.4%)	947 (53.6%)	
Female	1603 (48.3%)	1718 (51.7%)	
Area			<0.001
Rural	706 (53.9%)	603 (46.1%)	
Urban	1702 (45.4%)	2049 (54.6%)	
Sleep Quality Index	8.58 ± 1.77	7.76 ± 1.86	<0.001
Physical Activity	4.79 ± 2.10	3.94 ± 2.16	<0.001
Usage of Electronic Devices	4.30 ± 1.74	4.83 ± 2.03	<0.001
Nutrition Status	18.34 ± 3.02	16.89 ± 3.28	<0.001
Smoking			0.033
No	2317 (47.3%)	2578 (52.7%)	
Yes	114 (56.7%)	87 (43.3%)	
Drinking			0.654
No	1329 (47.1%)	1491 (52.9%)	
Yes	1102 (48.4%)	1174 (51.6%)	
Losing Weight or Not			0.060
No	1196 (49.3%)	1229 (50.7%)	
Yes	1233 (46.3%)	1430 (53.7%)	

^a^Total may not add up to 5100 due to missing data

**Fig. 1:**
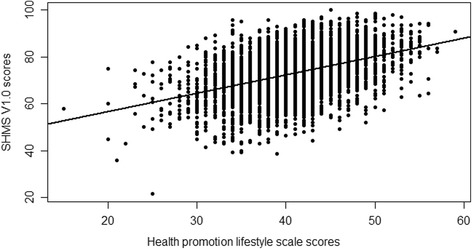
Scatter Plot of Suboptimal Health Measurement ScaleV1.0 Scores and Health Promotion Lifestyle Scale Scores

Figure 1 is the scatter diagram of the health status scores and health promotion lifestyle scale scores. It is easy to see from the figure that the SHMS V1.0 scores and HPLS scores have a positive correlation. The Pearson correlation coefficient is 0.4435 (*p* < 0.001).

### Multivariate logistic regression analyses of SHS

The multivariate logistic regression analyses results for SHS are shown in Table 4. Among them, “sleep quality index,” “physical activity,” “usage of electronic devices,” and “nutrition” are quantitative variables, while the rest are qualitative variables. After controlling for age group, gender, and areas, SHS was associated with sleep quality (aOR = 0.650, 95%CI = 0.612–0.690), physical activity (aOR = 0.889, 95%CI = 0.845–0.933), habits of using electronic devices (aOR = 1.066, 95%CI = 1.013–1.121), nutrition (aOR = 0.868, 95%CI = 0.864–0.908), smoking (aOR = 1.824, 95%CI = 1.195–2.755) and weight loss (aOR = 1.255, 95%CI = 1.043–1.509). Students aged 20–24 years old or living in an urban area were more likely to be classified in the healthy status group. The students with lower scores in sleep quality were more likely to be classified with SHS. Students who take part in less physical activity were more likely to be included in the SHS group. And students who spend more time on electronic devices were more likely to be categorized with SHS. Additionally, smoking was a risk factor of SHS, and the students with lower scores in nutrition were more likely to be grouped into SHS. Students who had lost weight were also more likely to be classified as having SHS.

**Table 4:** Logistic Regression Model for Health-Related Factors and Health Status

Health-Related Factors	Estimate	*P*	aOR	95%CI for aOR	
				Lower	Upper
Gender (male as a control)					
Female	0.030	0.786	1.030	0.831	1.280
Area (rural as a control)					
Urban area	−0.250	0.015	0.779	0.638	0.953
Age Group (15–19 as a control)					
20–24	−0.223	0.028	0.801	0.657	0.975
25 and above	0.073	0.614	1.075	0.808	1.423
Sleep Quality Index	−0.431	<0.001	0.650	0.612	0.90
Physical Activity	−0.118	<0.001	0.889	0.845	0.933
Usage of Electronic Devices	0.064	0.014	1.066	1.013	1.121
Nutrition	−0.142	<0.001	0.868	0.864	0.908
Smoking (no as a control)					
Yes	0.601	0.005	1.824	1.195	2.755
Drinking (no as a control)					
Yes	−0.034	0.735	0.966	0.792	1.177
Losing Weight (no as a control)					
Yes	0.227	0.016	1.255	1.043	1.509

## Discussion

The main purpose of this study is to better understand current situations of suboptimal health status in college freshmen and the factors affecting the health status of university students, with a particular emphasis on exploring the impact of electronic product usage on SHS. In our study, we found that the prevalence rate of SHS was 47.6% (2481/5344). This result is similar to those of other studies conducted in China [10, 14]. Although the prevalence of SHS is high, there has not objective clinical diagnostics for SHS so far. A number of SHS questionnaires have been established and evaluated in China, including the Multidimensional Sub-Health Questionnaire of Adolescents (MSQA) which is aimed at adolescents and the Suboptimal Health Status Questionnaire-25 (SHSQ-25) which is targeted at physiological and psychological SHS [34, 35]. The SHMS V1.0, which we used in our study, is a standardized questionnaire [33] used to assess respondents’ health status, and it contains a multidimensional, self-report symptom inventory. As freshmen enter a university, a number of students may have some physical, psychological, and social problems. Hence, for assessing the overall health status of students, the SHMS V1.0 is a suitable scale.

In our study, we found that some social demographic characteristics were associated with health status, such as age and area (Table 4). More students aged 15–19 years old were classified with SHS than those aged 20–24 years old. This result is not consistent with other studies, which found that younger adolescents had better health statuses [36]. We believe the main reason for this is that most of the students in the 15- to 19-year-old age group have just taken the college entrance examination. They’ve suffered from huge pressure to get good scores in order to enter an excellent college. Many students will sit and study all day, seldom going outdoors to exercise and sometimes paying no attention to their daily diet and nutrition, which results in harm to their health. We also found that students from rural areas had higher rates of SHS than those from urban areas. Students from rural areas often find that living environments when they enter college, usually located in a big city, are much different from those of their hometowns. They have to adapt in a short period of time and are more easily influenced by academic pressure or peer pressure, which could make them more prone to developing SHS [37].

We also found that some lifestyle behaviors were associated with health status, such as sleep quality, physical activity, the use of electronic equipment, nutrition, and losing weight. Poor sleep quality is positively associated with SHS. According to existing research, most young people need nine hours of restful sleep each night [38]. However, for a number of reasons, many school-aged children often get less than the recommended number of hours of sleep. Sleep quality has important implications for cognitive outcomes, mental health, physical health, work performance, and safety [39]. A mental health survey conducted by Gu et al. of 11,618 residents aged 18 years or older revealed that the rate of poor sleep quality in residents with mental disorders was 6.51 times higher than that of those without mental disorders [40]. Sleep-deprived individuals suffer from negative moods [41], are more likely to experience distress [42], are more likely to experience obesity [43], and are at a greater risk for coronary heart disease [44]. So poor sleep quality tends to lead to SHS.

Much existing evidence suggests that physical activity is associated with numerous health benefits [45, 46]. In general, the association of physical activity with mental health in young people is evident [47]. It has also been demonstrated that physical activity can influence the mental health of college students [48]. Our study found a significant relationship between low physical activity and SHS among college freshmen. Thus, it can be said that increased physical activity is helpful for college students, whose brains are highly plastic [49]. Possible mechanisms include an increase in serotonin or other neurotransmitters associated with the “endorphin effect” of alleviating negative feelings.

One main purpose of our study is to explore the relationship between electronic device usage and SHS. We found that overusing electronic equipment was positively associated with SHS. Electronic devices are ubiquitous among modern youth and are frequently used. Most students use electronic equipment to surf the Internet, and the negative impacts of uncontrolled Internet use have been a source of concern among researchers. Internet overuse has, in fact, been found to have negative impacts on psychological well-being, as well as social, occupational, academic, marital, and interpersonal relations [26, 50]. The EPIC Norfolk study showed that each 1-h/day increase in TV time was associated with increased hazard of all-cause and cardiovascular [51]. A dose response relationship was found between time using electronic equipment and psychological symptoms like poor appetite, loneliness, sleeping difficulties, sadness and hopelessness [52]. A study of 4747 college students in 2013 found that high screen time was associated with increased risks of mental health problems [53]. A similar relationship was also reported for neurological symptoms like dizziness, tremors, headache and stomach aches [54]. The studies above show the strong relationship between electronic equipment and health. Possible underlying mechanisms for the adverse health effects are complex. One of the ways that using electronic equipment has been hypothesized to influence health is by displacing time that could otherwise have been used for physical activity [51]. Another possible reason is that overusing electronic equipment is highly correlated with increased metabolic risk [55], and metabolic risk is associated with poor health [56]. So it can be said that more moderate use of electronic devices can improve the health status of college students.

We further found that poor nutrition status was associated with SHS among college freshmen. Many studies have previously found that poor nutrition status can seriously damage health [14, 17]. Chen et al. conducted a cross-sectional survey within a clustered sample of 24,159 individuals aged 12–80 years old during 2012–2013 in southern China [14]. This survey found that irregular breakfast-eating habits were related to an increased risk of SHS, and increased breakfast-eating frequency could contribute to lowering the prevalence of SHS in southern China. In our study, we found that losing weight was also highly correlated with SHS. Many students, especially girls, utilized diet pills, excessive dieting, and/or fasting to lose weight. A health survey conducted among 1629 students in 2009 found that unhealthy weight-reducing behavior was the main risk factor for SHS [57]. Previous studies have shown that insulin sensitivity can be reduced in response to pre-loading through excessive dieting, thus disturbing lipid profiles [58]. Further, excessive dieting and fasting can lead to deleterious metabolic and endocrine-related variation via the disruption of daily energy intake and upregulation of appetite. [59]. And weight loss over the long term can lead to malnutrition, which is harmful to one’s health. So emphasizing good nutrition status and healthy weight-reducing behaviors will benefit college students in terms of their health status.

SHS is an intermediate state between disease and health and is often medically undiagnosed. Students with diseases may worry about their health status and take some approaches to improve their symptoms, such as changing their poor lifestyle behaviors. SHS students, on the other hand, usually are not diagnosed. So they do not pay attention to their lifestyle behaviors, which leads to continuing health loss. Therefore, it is important to focus attention on SHS and lifestyle factors that threaten the health of young people. Prevention and intervention strategies aimed at SHS are effective approaches to improve health outcomes, the prevention of diseases, and the treatment of early-stage illnesses.

## Limitations

Some limitations for this study should be noted. First, this was a cross-sectional survey, which did not allow us to assess causality or the directionality of relationships. Second, all information was obtained from self-reported questionnaires, which could result in potential information bias. Also, we could not get some information, such as genetics, economic status, and so on which may be associated with SHS. Third, although the presence of poor sleep quality as well as suboptimal health status were assessed by standardized questionnaires, these measures are not equivalent to clinical diagnoses, thus future studies with diagnostic interviews should be used. Finally, the results may not represent all Chinese young adults, because all the participants who were general healthy and well-educated came from one large Chinese university and there was a big gender difference with 65% female which is higher than statistical data (52.11%) from Ministry of Education of the People’s Republic of China [60].

## Conclusion

Through this research into Chinese college freshmen, we found that poor lifestyle behaviors were significantly positively associated with SHS. In particular, the overuse of electronic devices is one of underlying causes of SHS. Focusing on these lifestyle factors, actions were recommended to improve the health status of this population.

## Additional file

Additional file 1: English copy of the questionnaire. (DOCX 24 kb)

## Abbreviations

CACM: China Association of Chinese Medicine
CIs: Confidence intervals
HPLP: Health Promoting Lifestyle Profile
HPLS: Health promotion lifestyle scale
OR: Odds ratios
SHMS V1.0: Suboptimal Health Measurement ScaleV1.0
SHS: Suboptimal health status
SQI: Sleep quality index
WHO: World Health Organization
