# Supplementary material for: Association between lifestyle factors and suboptimal health status among Chinese college freshmen: a cross-sectional study
Source: BMC Public Health. 2018 Jan 5;18:105. doi: 10.1186/s12889-017-5002-4 (PMC5755159; doi:10.1186/s12889-017-5002-4)
Supplement: Additional file 1: — English copy of the questionnaire. (DOCX 24 kb) [file 12889_2017_5002_MOESM1_ESM.docx]

No.______

**The Survey of Health-Related Behaviors among College Students**

Hello! I am an interviewer from the School of Statistics of Renmin University of China. We are conducting a research to get to know the situation of health-related behaviors among college students within Renmin University of China. The investigation process may take you about 30 minutes. We don’t record any personal information and we will keep all the information you provide as secret. Thank you for your support!

Note: Please fill in the questionnaire by yourself. Please fill in the number of the option you chose, such as "①". In the end of physical examination, please give your questionnaire to staff, thank you!

[Questionnaire processing record]

(Do not need to fill here)

Interviewer (signature): __________

First check (signature): _________

Second check (signature): __________

Inputer (Signature): __________

**Part one: Characteristics**

1. Your gender: __________ 1. Male 2. Female
2. Your student type: __________

1. Bachelor’s candidates 2. Master’s candidates 3. Doctoral candidates

1. Your school: __________
2. Your place of born: __________
3. Your date of born: __________
4. Your ethnicity: __________
5. The area you come from: __________ 1. Urban 2. Rural

**Part two: Lifestyle behaviors assessment**

The following questions focus on the past 6 months.

1. When do you usually go to bed? __________
2. How long (in minutes) does it take you to fall asleep each night? __________
3. What time do you usually get up in the morning? __________
4. How many hours of actual sleep do you get at night? __________ (non-bedridden time)
5. Do you play with an electronic device such as a mobile phone or a tablet computer after you go to bed at night?

1. Yes 2. No (please skip to question 7)

1. How long do you spend playing electronic devices such as a mobile phone or a tablet computer after going to bed each night?

1. <15 mins 2. 15-30 mins 3. 30-60 mins 4. >60 mins

1. How often do you have trouble falling asleep (cannot sleep within 30 minutes)?

1. 0 2. <1/week 3. 1-2/week 4. >3/week

1. How would you rate your sleep quality overall?

1. Very bad 2. Fairly bad 3. Fairly good 4. Very good

1. How often do you exercise each week (including running, swimming, playing, yoga, etc.)?

1. Never (please skip to question 11) 2. 1-2 times/week 3. 3-5 times/week 4. >5times/week

1. How long do you exercise per day (including running, swimming, playing, yoga, etc.)?

1. <15 mins/day 2. 15-30 mins/day 3. 30-60 mins/day 4. >60 mins/day

1. How long do you walk on average each day?

1. <15 mins/day 2. 15-30 mins/day 3. 30-60 mins/day 4. >60 mins/day

1. How long do you sit down on average each day?

1. <15 mins/day 2. 15-30 mins/day 3. 30-60 mins/day 4. >60 mins/day

1. How long do you watch TV on average each day?

1. never 2. <1 hour/day 3. 1-2 hours/day 4. 3-4 hours/day 5. >5 hours/day

1. How long do you use your computer or mobile device for entertainment (games, videos, etc.) on average each day?

1. never 2. <1 hour/day 3. 1-2 hours/day 4. 3-4 hours/day 5. >5 hours/day

1. Do you smoke?

1. no 2. Yes

1. Do you drink?

1. no 2. Yes

1. How often do you eat breakfast each week?

1. 0 2. 1-2 times 3. 3-4times 4. 5-6 times 5.7 times

1. How often do you eat fruits each week?

1. 0 2. 1-2 times 3. 3-4 times 4. 5-6 times 5. 7 times

1. How often do you eat vegetables (such as broccoli, carrot, lettuces, and so on) each week?

1. 0 2. 1-2 times 3. 3-4 times 4. 5-6 times 5. 7 times

1. How often do you eat fried food (such as fried chicken, French fried, and so on) each week?

1. 0 2. 1-2 times 3. 3-4 times 4. 5-6 times 5. 7 times

1. How often do you eat fish each week?

1. 0 2. 1-2 times 3. 3-4 times 4. 5-6 times 5. 7 times

1. How often do you eat eggs each week?

1. 0 2. 1-2 times 3. 3-4 times 4. 5-6 times 5. 7 times

1. How often do you drink milk, yogurt or other dairy each week?

1. 0 2. 1-2 times 3. 3-4 times 4. 5-6 times 5. 7 times

1. How often do you drink sodas each week?

1. 0 2. 1-2 times 3. 3-4 times 4. 5-6 times 5. 7 times

1. Do you have regular meals?

1. Very regular 2. General 3. No

26. Have you try to lost weight?

1. No 2. Yes

**Part three: Suboptimal health status evaluation**

The following questions focus on the past 6 months, please answer your own situation at that time.

1. How about your appetite?

1. Very bad 2. Bad 3. General 4. Good 5. Very good

1. How was your sleep?

1. Very bad 2. Bad 3. General 4. Good 5. Very good

1. Are you satisfied with your hair growth? (eg, early white hair, yellow hair or hair loss, etc.)

1. Never 2. Occasionally 3. Sometimes 4. Constantly 5. Always

1. Do you suffer from palpitations, chest tightness, or shortness of breath?

1. Never 2. Occasionally 3. Sometimes 4. Constantly 5. Always

1. Do you suffer from gastrointestinal discomfort? (eg, acid reflux, belching, nausea, abdominal pain, bloating, diarrhoea, constipation, etc.)

1. Never 2. Occasionally 3. Sometimes 4. Constantly 5. Always

1. Do you suffer from abnormal urine? (eg, dark urine, dysuria, oliguria, urinary frequency, nocturia, etc.)

1. Never 2. Occasionally 3. Sometimes 4. Constantly 5. Always

1. Do you suffer from head discomfort? (eg, dizziness, headache, heavy head, etc.)

1. Never 2. Occasionally 3. Sometimes 4. Constantly 5. Always

1. Are you suffering from eye discomfort? (eg, soreness, dryness, more tears, fuzzy, fatigue and more bloodshot eyes, etc.)

1. Never 2. Occasionally 3. Sometimes 4. Constantly 5. Always

1. Do you suffer hearing system abnormalities? (eg, tinnitus, hearing loss, earache, etc.)

1. Never 2. Occasionally 3. Sometimes 4. Constantly 5. Always

1. Do you have difficulty with your knees or with bending over?

1. No 2. Little 3. Some 4. Hard 5. Very hard

1. Do you have any difficulty in climbing 3–5 floors?

1. No 2. Little 3. Some 4. Hard 5. Very hard

1. Do you have any difficulty in walking 1500 m?

1. No 2. Little 3. Some 4. Hard 5. Very hard

1. Could the fatigue be alleviated by rest?

1. Never 2. Occasionally 3. Sometimes 4. Constantly 5. Always

1. Do you have enough energy to cope with everyday life, work and learn?

1. Never 2. Occasionally 3. Sometimes 4. Constantly 5. Always

1. You think you are in what physiological (physical) health status?

1. Health 2. Sub-health 3. Mild disease 4. Moderate disease 5. Severe disease

1. Do you have confidence?

1. Never 2. Little 3. Some 4. Much 5. quite

1. Are you satisfied with your living conditions?

1. Never 2. Little 3. General 4. Good 5. Very good

1. Are you optimistic about the future?

1. Never 2. Little 3. Some 4. Much 5. Quite

1. Are you feeling happy?

1. Never 2. Occasionally 3. Sometimes 4. Constantly 5. Always

1. Do you feel nervous?

1. Never 2. Occasionally 3. Sometimes 4. Constantly 5. Always

1. Do you experience bad moods or depression?

1. Never 2. Occasionally 3. Sometimes 4. Constantly 5. Always

1. Do you feel insecure?

1. Never 2. Occasionally 3. Sometimes 4. Constantly 5. Always

1. Do you have no reason to feel afraid?

1. Never 2. Occasionally 3. Sometimes 4. Constantly 5. Always

1. Do you feel lonely?

1. Never 2. Occasionally 3. Some 4. Much 5. Quite

1. Are you sensitive or suspicious?

1. Never 2. Occasionally 3. Sometimes 4. Constantly 5. Always

1. How is your memory?

1. Very bad 2. Bad 3. General 4. Good 5. Very good

1. What about your ability to think and solve problems?

1. Very bad 2. Bad 3. General 4. Good 5. Very good

1. How is your psychological health (eg, emotional, cognitive ability) status?

1. Health 2. Sub-health 3. Mild disease 4. Moderate disease 5. Severe disease

1. Can you appropriately deal with unhappy events in your life, work and school?

1. Never 2. Occasionally 3. Sometimes 4. Constantly 5. Always

1. Are you satisfied with your social relationships?

1. Never 2. Occasionally 3. Sometimes 4. Constantly 5. Always

1. Are you satisfied with your performance in your life, work and school?

1. Never 2. Occasionally 3. Sometimes 4. Constantly 5. Always

1. Can you quickly adapt to new living, working and learning environments?

1. Never 2. Occasionally 3. Sometimes 4. Constantly 5. Always

1. Do you always keep in touch with friends and family (eg, visits, phone calls, other communications)?

1. Never 2. Occasionally 3. Sometimes 4. Constantly 5. Always

1. Do you have friends to share your happiness and sadness?

1. No 2. Seldom 3. Several 4. Some 5. Many

1. Do you have many colleagues, classmates, neighbours, relatives or friends close to you?

1. No 2. Seldom 3. Several 4. Some 5. Many

1. When you need help, would your family, colleagues or friends provide physical or emotional support or help?

1. Never 2. Occasionally 3. Sometimes 4. Constantly 5. Always

1. When you are in trouble, would you seek support and help from others?

1. Never 2. Occasionally 3. Sometimes 4. Constantly 5. Always

1. What is the status of your social health (eg, interpersonal relationships, social interactions)?

1. Health 2. Sub-health 3. Mild disease 4. Moderate disease 5. Severe disease

1. What is the status of your health (including physiological, psychological and social aspects)?

1. Health 2. Sub-health 3. Mild disease 4. Moderate disease 5. Severe disease

**Part four: History of disease**

1. Have you ever been diagnosed with tuberculosis?

1. Yes 2. No (please skip to question 4)

1. When were you diagnosed with tuberculosis?

1. Within 6 months 2. Beyond 6 months

1. Is your tuberculosis healed now?

1. Yes 2. No 3. I don’t know

1. Have you ever been diagnosed with anemia?

1. Yes 2. No (please skip to question 7)

1. When were you diagnosed with anemia?

1. Within 6 months 2. Beyond 6 months

1. Is your anemia healed now?

1. Yes 2. No 3. I don’t know

1. Have you ever been diagnosed with myopia?

1. Yes 2. No (please skip to question 9)

1. When were you diagnosed with myopia?

1. Before junior middle school 2. Junior middle school 3. High school 4. After high school

1. Have your parents ever been diagnosed with myopia?

1. Both yes 2. One 3. Both no

1. In past 6 months, how many times do you catch a cold on average per month?

1. 0 2.1 times 3.2-3 times 4.>4 times

**Thanks for your cooperation!**
